# Diagnosis of *Pneumocystis jirovecii* pneumonia with serum cell-free DNA in non-HIV-infected immunocompromised patients

**DOI:** 10.18632/oncotarget.18037

**Published:** 2017-05-20

**Authors:** Dong Wang, Yang Hu, Ting Li, Heng-Mo Rong, Zhao-Hui Tong

**Affiliations:** ^1^ Department of Respiratory Medicine and Critical Care Medicine, Beijing Institute of Respiratory Medicine and Beijing Chao-Yang Hospital, Capital Medical University, Beijing, 100020, China

**Keywords:** cell-free DNA, immunocompromised patients, Pneumocystis jirovecii, polymerase chain reaction, serum

## Abstract

Conventional respiratory tract specimens, such as bronchoalveolar lavage (BAL) fluid and induced sputum for diagnosing *Pneumocystis jirovecii* pneumonia (PCP) in immunocompromised patients are difficult to obtain. Besides, bronchoscopy is an invasive procedure that carries the risk of causing rapidly progressive respiratory insufficiency. By contrast, serum cell-free DNA (cfDNA) is easy to obtain and has been proven useful in diagnosing cancer, pregnancy associated complications, parasite infection and sepsis. In this study, we performed quantitative polymerase chain reaction (qPCR) to assess the diagnostic efficiency of using serum cfDNA, BAL fluid, and sputum DNA for PCP. Seventy-one patients (35 PCP patients and 36 non-PCP patients) were enrolled according to the clinical PCP diagnostic criteria. The sensitivity, specificity, positive predictive value, and negative predictive value of PCR using serum cfDNA were 68.6% (95% CI, 50.7–83.1), 97.2% (95% CI, 85.5–99.9), 96.0%, and 76.1%, respectively. PCR using BAL fluid and sputum had a high sensitivity (97.1% and 91.4%, respectively) but relatively low specificity (86.1% and 86.1%, respectively). The combination of the sputum PCR OR serum cfDNA PCR yielded a sensitivity of 97.1%.These results indicated that serum cfDNA might be a valuable method in PCP diagnosis.

## INTRODUCTION

*Pneumocystis* Pneumonia (PCP) is a prevalent opportunistic fungus infection in immunocompromised patients, such as those with HIV infection, organ transplantation, malignancies, and connective tissue diseases [1–3]. Because of the use of highly active antiretroviral therapy, the incidence of PCP has decreased substantially in HIV-infected patients. However, in non-HIV-infected patients undergoing immunosuppressive therapies, whether with corticosteroids, monoclonal antibody or cytokine inhibitors, the morbidity and mortality of PCP in immunocompromised patients has remained high in recent years [4–7].

As an extracellular pathogen that is usually found in the alveolar cavity, *Pneumocystis* cannot readily be cultured in the laboratory. The gold standard method for the diagnosis of PCP mainly relies on microscopic detection for cysts in respiratory specimens; that is not sensitive enough [8]. Polymerase chain reaction (PCR) has been reported as a useful tool to assess *Pneumocystis* infection by detecting specimens from the respiratory tract. PCR on bronchoalveolar lavage (BAL) fluid and sputum is more sensitive than microscopic detection [9–11]. Nevertheless, in non-HIV PCP infected immunocompromised patients, operating bronchoscopy is usually difficult due to the underlying risk of rapidly progressive respiratory insufficiency [1]. Besides, patients with PCP often present with a non-productive cough, and induced sputum is not easy to obtain. Thus, a non-invasive method for *Pneumocystis* detection with high sensitivity and specificity is urgently required.

Cell-free DNA (cfDNA) is the fragments of DNA found extracellularly in different body fluids and tissues, mainly in circulating blood. Recent studies of cfDNA detection on serum, urine, and saliva samples have revealed cfDNA to perform satisfactorily in the diagnosis of parasitic infections [12]. The objective of this study is to reveal the diagnostic value of serum cfDNA through the PCR method in PCP, when compared with Grocott-Gomori methenamine silver (GMS) staining, sputum, and BAL fluid PCR.

## RESULTS

### Patient characteristics

**Figure 1:**
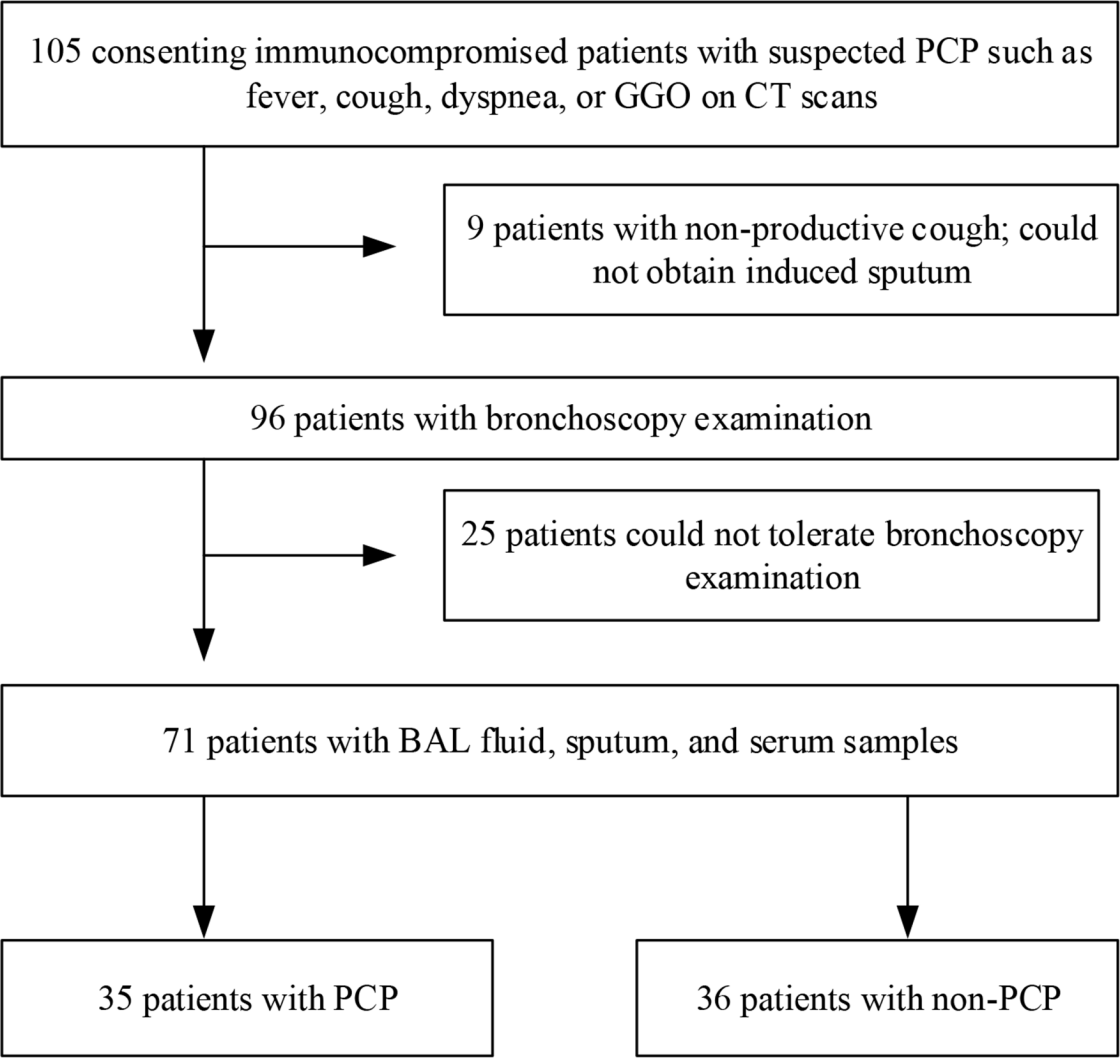
The screening and enrollment process of the study subjects

One hundred and five immunocompromised patients were recruited for this study from January 2015 to November 2016. Thirty-four of these were excluded due to either unavailable induced sputum or infeasible bronchoscopy procedure. As a result, 71 patients were enrolled; of these, 35 were clinically diagnosed with PCP while 36 had a non-PCP diagnosis (Figure 1). Among the 35 patients with clinical PCP, 18 were defined as PCP with positive GMS staining (10 with positive BAL fluid staining, 4 with positive sputum staining, and 4 with positive BAL fluid and sputum staining). Among the 36 non-PCP patients, 1 was diagnosed with pulmonary embolism, 1 was diagnosed with lung cancer, 2 were diagnosed with interstitial pneumonia, and 32 were diagnosed with as severe pneumonia (9 viral pneumonia, 17 bacterial pneumonia, and 6 fungal pneumonia, other than PCP).

The underlying diseases of the 71 patients could be divided into 3 groups: solid organ transplantation (5 with liver transplantation and 17 with kidney transplantation); malignancies (3 with leukemia, 2 with lymphoma, and 9 with solid tumors); immunosuppressed diseases (*n* = 35) (either experiencing some kind of immunodeficiency, or receiving either immunosuppressive, or cytotoxic medication) (Table 1).

**Table 1:** Demographic and baseline cinical characteristics of enrolled patients with immunocompromised diseases

Clinical characteristics	Total (*n* = 71)	PCP (*n* = 35)	Non-PCP (*n* = 36)	*P* value
**Age^†^**	51.7 ± 14.4	54.5 ± 13.7	48.9±14.7	0.150
**Male**	50 (70.4%)	24 (68.6%)	26(72.2%)	0.736
**Underlying diseases**				
** Transplantation**	22 (31.0%)	15 (42.9%)	7 (19.4%)	0.033
Liver transplantation	5 (7.0%)	3 (8.6%)	2 (5.6%)	0.974
Kidney transplantation	17 (23.9%)	12 (34.3%)	5 (13.9%)	0.044
**Malignancies**	14 (19.7%)	3 (8.6%)	11 (30.6%)	0.020
Leukemia	3 (4.2%)	1 (2.9%)	2 (5.6%)	0.980
Lymphoma	2 (2.8%)	0 (0%)	2 (5.6%)	0.493
Solid tumor	9 (12.7%)	2 (5.7%)	7 (19.4%)	0.167
**Immunosuppressed diseases**	35 (49.3%)	17 (48.6%)	18 (50.0%)	0.904
Chronic kidney disease	13 (18.3%)	9 (25.7%)	4 (11.1%)	0.112
Interstitial pneumonia	2 (2.8%)	1 (2.9%)	1 (2.8%)	1.000
Autoimmune diseases	20 (28.2%)	7 (20.0%)	13 (36.1%)	0.131
**Symptoms**				
Dyspnea	56 (78.9%)	30 (85.7%)	26 (72.2%)	0.164
Cough	62 (87.3%)	29 (82.9% )	33 (91.7%)	0.448
Sputum	35 (49.3%)	13 (37.1% )	22 (61.1%)	0.043
Fever	65 (91.5%)	32 (91.4%)	33 (91.7%)	0.696
GGO	44 (62.0%)	30 (85.7%)	14 (38.9%)	< 0.001

Values are presented as number (%).

^†^ Values are presented as mean ± standard deviation.

### Diagnostic utility of GMS staining, BAL fluid, and sputum PCR

When using defined diagnosis criteria, there were 18 PCP patients. The diagnostic consistency of PCR with GMS staining is summarized in Table 2. In the 18 defined PCP patients, BAL fluid PCR was positive in all patients, while sputum PCR was positive in 17 patients. When using GMS staining as the gold standard for diagnosing PCP, the sensitivity of PCR on BAL fluid and sputum was 100% (95% CI, 81.5–100) and 94.4% (95% CI, 72.7–99.9),respectively, and the specificity of these 2 methods was 60.4% (95% CI, 46.0–73.5) and 62.3% (95% CI, 47.9–75.2), respectively.

**Table 2:** Diagnostic performance of PCR in defined PCP patients

	GMS staining + (*n* = 18)	GMS staining − (*n* = 53)	Total (*n* = 71)
BAL fluid PCR +	18	21	39
BAL fluid PCR −	0	32	32
Sputum PCR +	17	20	37
Sputum PCR −	1	33	34
Serum PCR +	14	11	25
Serum PCR −	4	42	46

In the 35 clinical PCP patients, GMS staining, PCR on BAL fluid, and PCR on sputum was positive in 18, 34, and 32 cases, respectively (Table 3), When using clinical diagnosis criteria, the sensitivity of GMS, PCR on BAL fluid, and PCR on sputum was 51.4% (95% CI, 34.0–68.6), 97.1% (95% CI, 85.1–99.9), and 91.4% (95% CI, 76.9–98.2), respectively. The specificity of GMS staining, PCR on BAL fluid, and PCR on sputum was 100% (95% CI, 90.3–100), 86.1% (95% CI, 70.5–95.3), and 86.1% (95% CI, 70.5–95.3), respectively (Table 4).

**Table 3:** Diagnostic performance of GMS staining and PCR in clinical PCP patients

	Clinical PCP (*n* = 35)	Non-PCP (*n* = 36)	Total (*n* = 71)
GMS staining +	18	0	18
GMS staining −	17	36	53
BAL fluid PCR +	34	5	39
BAL fluid PCR −	1	31	32
Sputum PCR +	32	5	37
Sputum PCR −	3	31	34
Serum PCR +	24	1	25
Serum PCR −	11	35	46

**Table 4:** Diagnostic performance of GMS staining, BAL fluid PCR, sputum PCR, serum PCR, and the combinations of sputum PCR and serum PCR for PCP diagnosis

Test	Sen (%) 95% CI	Spe (%) 95% CI	PPV (%)	NPV (%)
GMS staining	51.4 (34.0–68.6)	100 (90.3–100)	100	67.9
BAL fluid PCR	97.1 (85.1–99.9)	86.1 (70.5–95.3)	87.2	96.9
Sputum PCR	91.4 (76.9–98.2)	86.1 (70.5–95.3)	86.5	91.2
Serum PCR	68.6 (50.7–83.1)	97.2 (85.5–99.9)	96.0	76.1
Sputum PCR				
AND serum PCR	62.9 (44.9–78.5)	100 (90.3–100)	100	73.5
Sputum PCR				
OR serum PCR	97.1 (85.1–99.9)	83.3 (67.2–93.6)	85.0	96.8

### Diagnostic utility of serum cfDNA PCR

When using defined diagnosis criteria, serum cfDNA PCR was positive in 14 patients. The sensitivity of serum cfDNA PCR was 77.8 % (95% CI, 52.4–93.6). There was no significant difference in comparison with BAL fluid PCR (*P* = 0.134) and sputum PCR (*P* = 0.249). However, the specificity of serum cfDNA PCR (79.3%, 95% CI, 65.9–89.2) was higher than was that using both BAL fluid PCR (*P* = 0.009) and sputum PCR (*P* = 0.039).

When using clinical diagnosis criteria, serum cfDNA PCR was positive in 24 patients. The sensitivity of serum cfDNA PCR (68.6%, 95% CI, 50.7–83.1) was lower than was that using BAL fluid PCR (*P* = 0.004) and sputum PCR (*P* = 0.043). The specificity of serum cfDNA PCR (97.2%, 95% CI, 85.5–99.9) showed no significant difference with BAL fluid PCR (*P* = 0.221) and sputum PCR (*P* = 0.221), although there was a higher trend.

### Diagnostic utility of combined serum cfDNA and sputum PCR

The sensitivity and specificity of combined PCR on sputum and serum testing among 71 patients are summarized in Table 4. The combination of positive sputum AND positive serum criteria yielded a high specificity (100%, 95% CI, 90.3–100) with a sensitivity, positive predictive value (PPV), and negative predictive value (NPV) of 62.9% (95% CI, 44.9–78.5), 100% and 73.5%, respectively. The combination of positive sputum OR positive serum had a high sensitivity (97.1%, 95% CI, 85.1–99.9) with a specificity, PPV, and NPV of 83.3% (95% CI, 67.2–93.6), 85.0 % and 96.8 %, respectively.

### Study summary of serum DNA in the diagnosis of PCP

In our study, we searched the papers related to serum DNA detection in the diagnosis of PCP [13–20]. We found that the serum quantity, DNA extraction methods, PCR methods, PCR target and the standard to define PCP diagnosis were quite different among these studies (Table 5). As a result, the diagnostic sensitivities ranged from 0% to 100%, while the diagnostic specificities ranged from 86.4% to 100%. Regardless of the differences in these studies, the pooled sensitivity and specificity of serum DNA detection were 78/141 (55.3%, 95% CI, 46.7–63.7) and 147/150 (98.0%, 95% CI, 94.3–99.6), respectively.

**Table 5:** Study summary of serum DNA in PCP diagnosis

Year	PCR method	PCR target	Sensitivity (%)	Specificity (%)	Reference
1992	C-PCR	DHFR	12/14 (85.7)	6/6 (100)	[13]
2012	qPCR	mt LSU rRNA	9/10 (90.0)	60/60 (100)	[14]
1998	N-PCR	ITSs	10/14 (71.4)	26/26 (100)	[15]
1996	N-PCR	mt LSU rRNA	0/15 (0)	-	[16]
1996	C-PCR	5S rRNA	10/10 (100)	-	[17]
1995	N-PCR	ITSs	27/27 (100)	18/18 (100)	[18]
		DHFR	2/20 (10.0)	18/18 (100)	
1997	C-PCR	mt LSU rRNA	1/13 (7.7)	19/22 (86.4)	[19]
1991	C-PCR	DHFR	7/18 (38.9)	-	[20]

Abbreviations: C-PCR, conventional PCR; N-PCR, nested PCR; DHFR, dihydrofolate reductase; ITSs, internal transcribed spacers.

## DISCUSSION

PCP is one of the most common pulmonary infection pathogens in immunocompromised patients. Laboratory methods for the diagnosis of PCP include etiological and molecular methods. Staining methods, including microscopy of the BAL fluid, sputum, or oropharyngeal wash, are the simplest methods used to confirm the diagnosis of PCP. However, the greatest disadvantage of the staining method is its low sensitivity [8, 21]. In our study, the sensitivity of staining is only 51.4%, partly due to the low *Pneumocystis* burden in non-HIV-infected patients [8] and the lack of experience of the observer and bronchoscopy operator. The low sensitivity of GMS staining resulted in a relatively small number of defined PCP cases in our study, thus leading to the low specificity of PCR when using GMS staining the gold standard.

Our results of PCR on BAL fluid and sputum show a high sensitivity (97.1% and 91.4%, respectively), which is consistent with other studies [22–24]. The specificity of PCR on BAL fluid and sputum are both 86.1% in our study, possibly due either to the presence of colonization of *Pneumocystis* in chronic lung disease patients [23, 25, 26] or to the presence of dead organisms. Considering that BAL fluid and sputum samples are difficult to obtain in some PCP patients, such as critically ill and those with a non-productive cough, alongside the unsatisfying specificity of PCR on BAL fluid and sputum, PCR detection on different blood compartments has been developed as a noninvasive procedure to improve the diagnosis of PCP [27].

cfDNA is the fragments of DNA found extracellularly in different body fluids, mainly in circulating blood. It has been known for a few years that cfDNA can be used for diagnosing cancer, pregnancy associated complications, and infectious diseases, such as *Plasmodium, Trypanosoma, Leishmania, Schistosoma, and Wuchereria spp.* infections [12, 28, 29]. In studies related to parasite infections, cfDNA PCR has a relatively low diagnostic sensitivity, mainly due to the inadequacy of DNA extraction methods, which leads to a reduction of substrate content in PCR assays [12]. In our study, when using GMS staining as the gold standard, the sensitivity of serum cfDNA PCR was no different to that of BAL fluid PCR and sputum PCR, while the specificity was much higher. When using clinical diagnosis of PCP as diagnostic criteria, the serum cfDNA PCR showed high specificity and PPV, which were both superior to PCR on BAL fluid and sputum. According to the above evidence, serum cfDNA PCR is a valuable PCP diagnostic method.

According to the previous studies, the diagnostic performance of PCR on serum DNA has shown conflicting results, with a sensitivity ranging from 0–100% [27, 30]. This might be related both to the serum quantities used for DNA extraction and to technical variations, including DNA extraction methods, PCR methods, and targets for detecting *Pneumocystis*. In those studies, Proteinase K-phenol-chloroform was the main serum DNA extraction method. However, in our study, we chose magnetic bead methods, which is an efficient method in serum cfDNA extraction. In addition, different *Pneumocystis* primers may affect the results of cfDNA PCR. We used the *Pneumocystis* heat shock protein (HSP) 70 gene as the target gene, while most of the previous studies used *Pneumocystis* mitochondrial large subunit ribosome RNA (mt LSU rRNA) gene in *Pneumocystis* detection. As proved by Hugett et al., the HSP70 gene has higher sensitivity and specificity in *Pneumocystis* detection than does the *Pneumocystis* mt LSU rRNA gene [31].

The combined specificity of “positive sputum AND positive serum” PCR is 100%, which is superior to the specificity of BAL fluid PCR, and this approach could be used to reduce the incidence of the false positive rate when used in clinical criteria. The combined sensitivity of “positive sputum OR positive serum” is as high as is the sensitivity of BAL fluid PCR detection. Consequently, to reduce the misdiagnosis rate, the combination of “positive sputum OR positive serum” should be used in patients who are highly suspected of having PCP with bronchoscopy intolerance.

There are some limitations in this study. First, as it is a single center study with a relatively small enrolled patient number, this may cause a selection bias. Although this could not be avoided, future studies will focus on validating our results in a multicenter setting. Second, since our aim is to compare the sensitivity and specificity of PCR using BAL fluid, sputum, and serum cfDNA in PCP diagnosis, the PCR test on *Pneumocystis* in this study was qualitative rather than quantitative, which is responsible for the failure in investigating the consistency of the *Pneumocystis* load in BAL fluid/sputum with it in serum. Third, cfDNA is unstable in the circulating blood and will be degraded by nucleases. According to a published study, the half-life of fetal cfDNA in the maternal blood circulation is around 16 minutes [32]. Similarly, a study by M. Fleischhacker et al. has shown that the half-life of circulating tumor DNA is only from 15 minutes to several hours [33]. For these reasons, we theorized that a rapid detection of the sample may improve the diagnostic performance.

In conclusion, PCR on both BAL fluid and sputum is highly sensitive in PCP diagnosis, while GMS staining and serum PCR are highly specific. In critically ill patients who cannot tolerate bronchoscopy, the combination of PCR positive on serum or positive on sputum implies a very high probability of PCP.

## MATERIALS AND METHODS

### Study population and sample collection

A prospective study that enrolled non-HIV immunocompromised patients suspected of PCP was conducted in Beijing Chao-Yang Hospital from January 2015 to November 2016. According to our published study [34], the immunocompromised status meets one of the following factors: treatment with immunosuppressants during the past 90 days; receipt of an allogeneic stem cell or solid organ transplant; chronic underlying disease or major operations; prolonged use of corticosteroids; and inherited severe immunodeficiency. Suspected PCP was defined as either having clinical symptoms such as cough, fever, and dyspnea, or having ground grass opacity (GGO) in high resolution CT scans. The study was approved by the Institutional Review Board of Beijing Chao-Yang Hospital, and written informed consent was obtained from all participants. Patients who either could not undergo bronchoscopy or failed to obtain sputum samples were excluded from the study. Demographic and medical data were collected from the enrolled patients. Peripheral blood, sputum (spontaneous or induced sputum), and BAL fluid under bronchoscopy were also collected from them. The sputum samples for PCR were collected more than once in the enrolled patients, but only the first result of sputum PCR was evaluated for its diagnostic value.

### Diagnosis of PCP

The following criteria were used for the diagnosis of PCP: (1) BAL fluid or sputum GMS staining yielded *Pneumocysits* cysts. (2) BAL fluid or sputum PCR yielded positive results in two replicates. The defined diagnosis was defined to meet the criteria (1). The clinical diagnosis was defined to meet the criteria (2).

### Samples processing

BAL fluid samples were centrifuged at 1,600 g for 5 min at 4°C. To avoid non-specific inhibition, no mucolytic agent was used for these samples. Sputum samples were first treated with equal volumes of pancreatin at 37°C for 15 min and then centrifuged at 1,600 g for 10 min. The resulting pellet was washed twice with phosphate buffer saline (PBS) and resuspended in 1 ml of PBS. Blood samples were centrifuged at 6,000 g for 10 min at 4°C. The serum was obtained and centrifuged at 16,000 g for another 10 min at 4°C.

### Staining for Pneumocystis jirovecii

A portion of resuspended pellet of sputum was used to make a smear for GMS staining, according to the manufacturer's instructions (Baso, Zhuhai, China). The stained smears were visualized under a microscope for *Pneumocystis* cysts.

### DNA extraction and qPCR

For BAL fluid and sputum samples, DNA was extracted from the resuspended pellet using a QIAamp DNA Mini kit (Qiagen, Hamburg, Germany), according to the manufacturer's instructions. For serum samples, cfDNA was extracted using the MagMAX^™^ Cell-Free DNA Isolation Kit (Thermo Fisher Scientific, Massachusetts, US), according to the instructions provided in the kit. DNA and cfDNA of a 151 bp fragment of the *Pneumocystis jirovecii* HSP70 gene were detected by qPCR. The forward primer was 5′-GCA GGA TTG AAT GTT TTA C-3′, the reverse primer was 5′-CCT CTT CGA TAG TTA ATA ACG-3′, and the TaqMan probe was 5′-FAM-CAA TGA ACC TAC AGC AGC AGC C-MGBNFQ-3′.

### Statistics analysis

Comparison of two independent samples was performed using a Mann–Whitney *U* test and chi-square tests, and a *P* value of less than 0.05 was considered statistically significant. The results of the diagnostic tests were described as sensitivity, specificity, PPV, and NPV. All analyses were calculated using the software 19.0 (SPSS Inc., Chicago, US) and MedCalc (MedCalc Software, Ostend, Belgium).
